# Decellularized Testicular Extracellular Matrix Scaffolds Support Mature Spermatogenesis: Impact of Donor Age and Transplantation Microenvironment

**DOI:** 10.3390/ijms27114828

**Published:** 2026-05-27

**Authors:** Jung-Hsiu Hou, How Tseng, Bo-Sheng Xiao, Yu-Chio Wang, Sung-Ming Weng, Chi-Huang Chen

**Affiliations:** 1 Division of Reproductive Medicine, Department of Obstetrics and Gynecology, Taipei Medical University Hospital, Taipei 110301, Taiwan; b101099044@tmu.edu.tw (J.-H.H.); d118114005@tmu.edu.tw (Y.-C.W.); 2 Graduate Institute of Medical Science, College of Medicine, Taipei Medical University, Taipei 110301, Taiwan; tsenghow@tmu.edu.tw; 3 Department of Biochemistry and Molecular Cell Biology, School of Medicine, College of Medicine, Taipei Medical University, Taipei 110301, Taiwan; 4 International Ph.D. Program for Cell Therapy and Regeneration Medicine, College of Medicine, Taipei Medical University, Taipei 110301, Taiwan; 5 Department of Obstetrics and Gynecology, School of Medicine, College of Medicine, Taipei Medical University, Taipei 110301, Taiwan; a04679@tmu.edu.tw (B.-S.X.); toin@tmu.edu.tw (S.-M.W.); 6 Graduate Institute of Clinical Medicine, College of Medicine, Taipei Medical University, Taipei 110301, Taiwan

**Keywords:** bioluminescence imaging, decellularized testicular extracellular matrix, fertility preservation, immature testicular tissue transplantation, microenvironment, regenerative medicine, spermatogenesis, testicular scaffold, tissue engineering

## Abstract

Immature testicular tissue transplantation is a promising strategy for restoring fertility in prepubertal boys undergoing gonadotoxic treatments, yet it faces challenges such as significant germ cell loss and poor graft survival due to the initial period without a blood supply. This study utilized an in vivo tissue-engineering platform involving decellularized testicular extracellular matrix to enhance graft stability and maturation. Immature testicular tissue from three-week-old transgenic mice was transplanted into age-matched recipient mice across four experimental groups: tissue co-transplanted with young decellularized matrix into a cleared testicular cavity, tissue with adult matrix in a cleared cavity, tissue transplanted alone in a cleared cavity, and tissue injected directly into an intact recipient testis. Graft growth was monitored longitudinally using bioluminescence imaging, and spermatogenesis was evaluated via histology and immunohistochemistry sixty-five days post-transplantation. The decellularization protocol successfully removed more than 98 percent of host deoxyribonucleic acid while preserving key matrix components. Grafts injected into intact testes showed the earliest bioluminescence peak but subsequently declined. Conversely, tissue co-transplanted with young decellularized matrix in a cleared cavity exhibited a delayed but significantly higher and more sustained peak compared to the group without a scaffold. The young matrix group appeared to provide more sustained support for spermatogenic progression, as reflected by a more stable increase and a prolonged spermatogenic peak over time. Evidence of advanced spermatogenic stages, including spermatids and sperm-like cells, was observed in all groups by day sixty-five. In conclusion, decellularized testicular matrix serves as a supportive bioactive scaffold that improves long-term graft stability, with outcomes significantly influenced by the age of the scaffold’s donor and the transplantation microenvironment.

## 1. Introduction

For prepubertal boys who require fertility preservation because of cancer therapy or other diseases, there is currently no clinically proven method to restore future fertility. Testicular tissue cryopreservation (TTC) is available as an experimental option, but the central challenge is how to use the cryopreserved tissue safely and effectively to recover spermatogenesis. Meanwhile, the use of TTC is increasing, with surveys of participating centers reporting that more than 3000 patients have undergone the procedure [1], and this number is expected to rise as access expands. To date, neither live birth nor complete functional spermatogenic restoration has been reported after the transplantation of cryopreserved human immature testicular tissue. Similarly, complete spermatogenesis has not yet been achieved through the human in vitro spermatogenesis of immature testicular tissue. Current fertility-restoration strategies under investigation include spermatogonial stem cell transplantation (SSCT), in vitro spermatogenesis (IVS), and immature testicular tissue transplantation (ITTT) [2].

Among these, ITTT is widely considered the approach closest to clinical translation. In ITTT, cryopreserved immature testicular tissue fragments (grafts) are transplanted back in vivo, where host vasculature and the endocrine milieu support tissue survival and maturation. The strongest evidence is the landmark report by Fayomi and colleagues in 2019 [3], who achieved a live birth in a nonhuman primate (rhesus macaque) model using sperm derived from matured transplanted tissue. However, since this report, the field has seen limited major clinical advances. ITTT is fundamentally challenged by massive germ cell loss and poor graft survival, primarily attributed to the hypoxia/reoxygenation injuries sustained during the avascular period preceding host revascularization [4]. ESHRE has issued good practice recommendations for testicular tissue cryopreservation in boys receiving gonadotoxic treatment, covering procedures such as biopsy and cryostorage [5]. Nevertheless, fertility restoration using cryopreserved testicular tissue, including autologous transplantation, remains experimental. Notably, an early clinical report of autologous transplantation in an adult showed graft survival with detectable blood flow but no mature sperm [6], highlighting that key transplantation conditions still require optimization, regarding early graft survival, implantation site details, and safety with respect to malignant cell contamination.

To address early graft loss and optimize the graft niche, our group previously investigated tissue-engineered scaffolds and found that a poly(L-lactic acid) (PLLA) fine scaffold produced superior transplantation outcomes [7]. Mechanistic analyses suggested that this benefit was driven by the high biomimicry of its nanofibrous architecture. Specifically, it markedly reduced scaffold stiffness, bringing the mechanical properties closer to those of native testicular tissue (approximately 4.77 kPa), while also recapitulating the fibrous network of the native extracellular matrix (ECM). These findings support the concept that a microenvironment with tissue-matched structure and compliance is critical for long-term graft survival and functional maturation, compatible with the previous studies [8].

Building on these results, we next focused on natural biomaterials, particularly ECM scaffolds generated by decellularization. Decellularized ECM scaffolds are a highly promising biological material for regenerative medicine because they efficiently remove immunogenic cellular components while preserving the nonimmunogenic, natural 3D organization and specific bioactive factors often lacking in artificial supports [9]. The ideal strategy is to use the decellularized ECM of the target tissue, as this ensures the most appropriate site-specific architectural, biomechanical, and molecular composition needed for the effective induction of functional tissue regeneration and restoration [10]. Accordingly, decellularized testicular ECM (dT-ECM) may be a promising strategy for mitigating ischemic injury and improving graft survival in ITTT.

**Figure 1:**
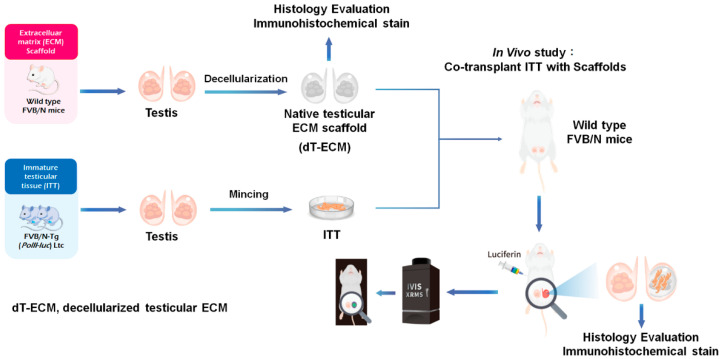
Schematic overview of the study design. Wild-type FVB/N testes were decellularized to generate decellularized testicular ECM (dT-ECM) scaffolds for histological and immunohistochemical characterization. Immature testicular tissue (ITT) was prepared by mincing testes from FVB/N-Tg(*PolII-luc*)Ltc transgenic donor mice and transplanted into wild-type FVB/N recipients either alone or with dT-ECM. Graft survival was monitored longitudinally by bioluminescence imaging (BLI), followed by endpoint histology and immunohistochemistry.

We hypothesized that dT-ECM provides a tissue-specific bioactive scaffold that enhances immature testicular tissue graft survival and spermatogenic progression, and that dT-ECM derived from younger donors offers more favorable support than adult dT-ECM. The primary objective of this study was to determine whether dT-ECM can support the survival and development of immature testicular tissue grafts. We further examined whether the donor age of the dT-ECM scaffold influences transplantation outcomes. By defining the efficacy and optimal conditions of dT-ECM, we aim to establish a durable tissue-engineering platform to advance fertility preservation for prepubertal boys. A schematic overview of the experimental design is shown in Figure 1.

## 2. Results

### 2.1. The Decellularization Method Successfully Removed Most of the DNA Content of the Donor Mice’s Testicular Tissue

**Figure 2:**
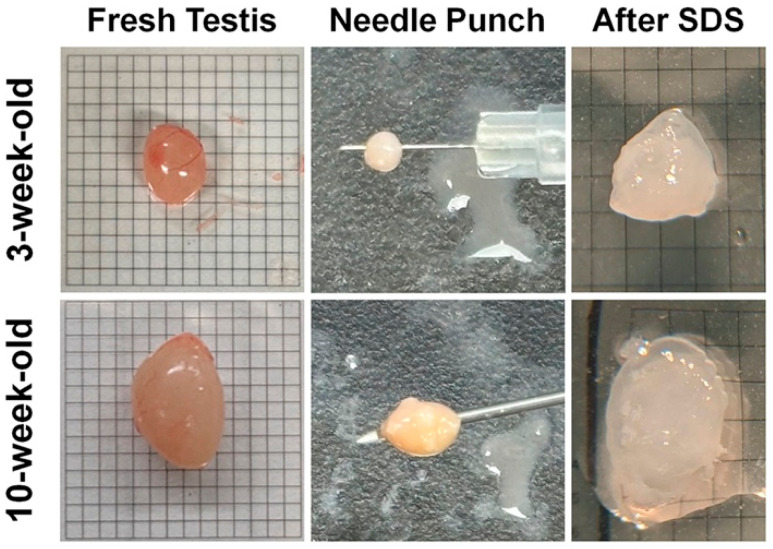
Gross appearance of testes during decellularization. Macroscopic photographs show wild-type mouse testes at key stages of the decellularization process, including fresh specimens, during one of the axial penetrations, and after SDS washing. The tissue progressively changes from a pink, flesh-like appearance to a white and mildly translucent scaffold.

**Figure 3:**
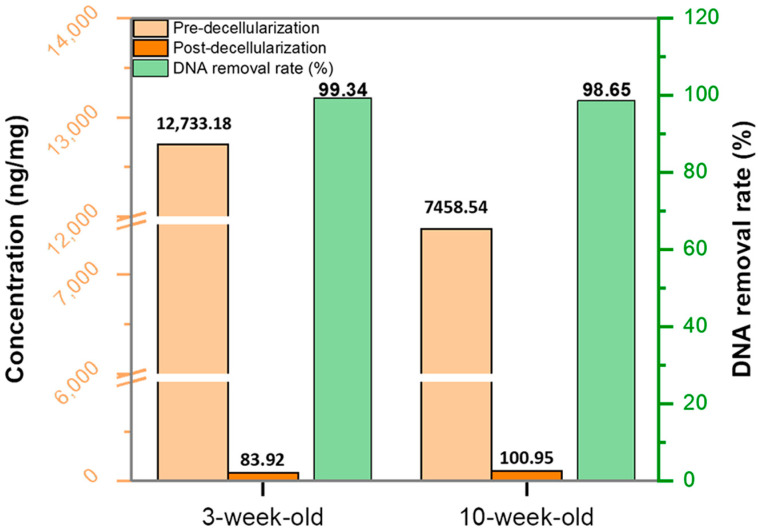
DNA content before and after decellularization. Residual DNA content (ng/mg) and DNA removal rate (%) are shown for 3-week-old and 10-week-old donor testes before and after decellularization. In both age groups, decellularization markedly reduced DNA content and achieved DNA removal rates greater than 98%, supporting effective decellularization.

Wild-type mouse testes were decellularized as described above. We first assessed macroscopic changes as an initial indicator of successful decellularization (Figure 2). Intact testes were perforated along 3 axes, and the tissue gradually changed from a pink, flesh-like appearance to a white and mildly translucent scaffold during the decellularization process. Residual DNA content was then quantified to assess decellularization efficiency (Figure 3). In the 3-week group, DNA content decreased from 12,733.18 ng/mg before decellularization to 83.92 ng/mg after decellularization, corresponding to a 99.34% DNA removal rate. In the 10-week group, the pre-decellularization DNA content was higher at 7458.54 ng/mg, while residual DNA after decellularization remained low at 100.9 ng/mg, yielding a 98.65% DNA removal rate. Overall, this protocol achieved highly efficient DNA removal in both age groups, with removal rates exceeding 98%.

### 2.2. The Characteristics of the Decellularized Testicular ECM Scaffold

**Figure 4:**
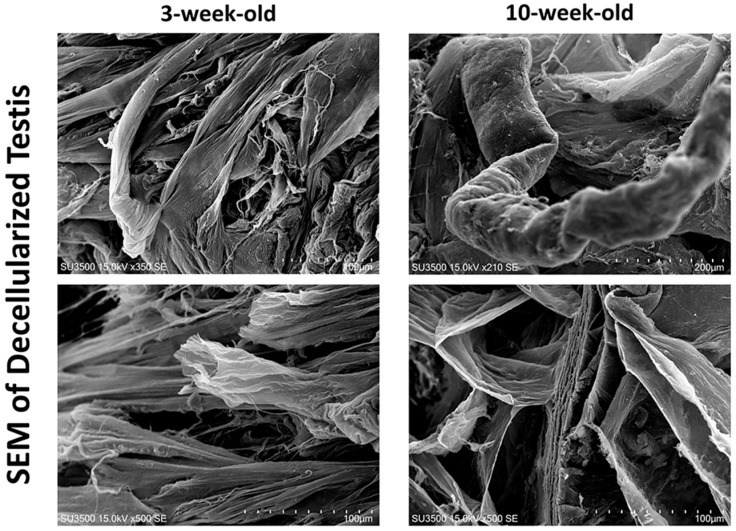
Scanning electron microscopy ultrastructure of dT-ECM derived from young and adult donors. Age-related architectural differences were preserved after decellularization, with seminiferous tubule diameters of approximately 35 µm in the 3-week group and approximately 95 µm in the 10-week group. No obvious residual cellular material was observed in either group.

Under scanning electron microscopy, the structural features of dT-ECM differed by donor age (Figure 4). The seminiferous tubule diameter was approximately 35 µm in the 3-week group and approximately 95 µm in the 10-week group, which is about 2 to 3 times larger than that of the 3-week group. In addition, no obvious residual cellular material was observed in either group. These findings indicate that testicular ECM architecture varies with donor age and that these age-related differences are preserved after decellularization. Therefore, dT-ECM derived from different donor ages should be considered distinct scaffolds.

**Figure 5:**
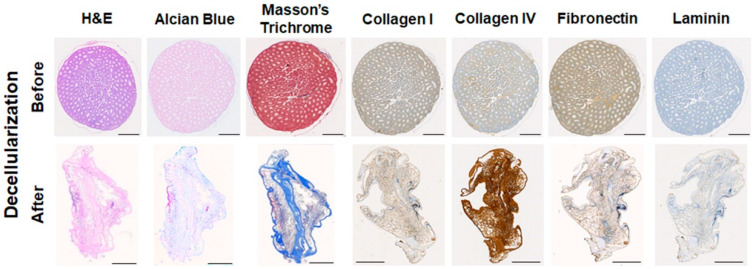
Histochemical staining and immunohistochemistry (IHC) of native testis and dT-ECM. Representative staining compares native testicular tissue before decellularization with dT-ECM after decellularization. H&E confirms removal of cellular components. Alcian blue shows retention of glycosaminoglycans, and Masson’s trichrome demonstrates preservation of collagen-rich matrix. IHC further confirms Collagen I, Collagen IV, Fibronectin, and Laminin, supporting the preservation of key ECM components after decellularization. Bar = 1000 μm.

Using histochemical staining and immunohistochemistry, we confirmed that decellularization effectively removed cellular components while preserving the bioactivity and structural characteristics of the native extracellular matrix (Figure 5). Alcian blue staining demonstrated the retention of glycosaminoglycans within the scaffold. Masson’s trichrome staining showed the preservation of collagen-rich matrix, and IHC further confirmed the presence of major structural collagens, including collagen I and collagen IV. In addition, key adhesive glycoproteins, fibronectin and laminin, remained detectable after decellularization. Collectively, the maintenance of these testis-specific ECM components indicates that dT-ECM retains both biochemical composition and tissue-relevant matrix features, which may provide mechanical and biochemical cues to transplanted cells and better recapitulate the native testicular niche.

### 2.3. Cyclic Changes Among the Groups

**Figure 6:**
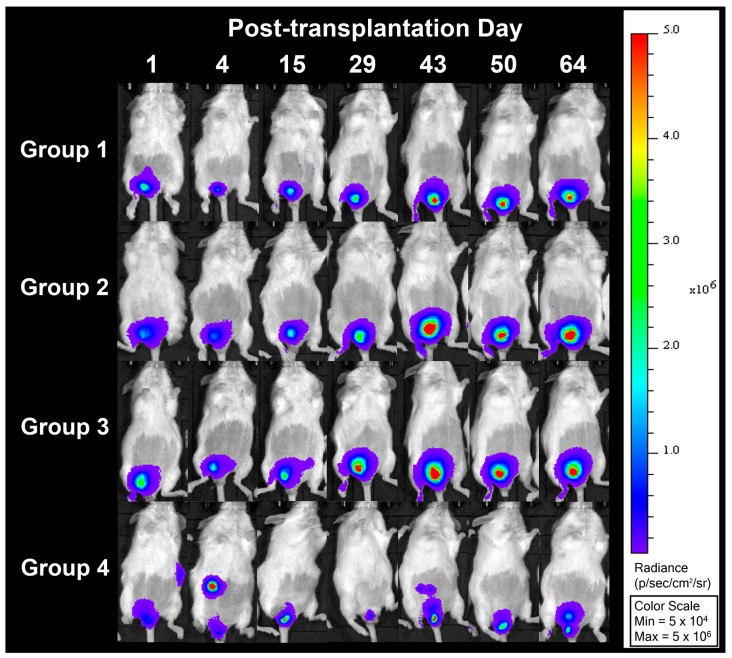
Longitudinal BLI after transplantation. Representative BLI images show engrafted testicular tissue signals across the four groups at Day 1, 4, 15, 29, 43, 50, and 64. Images illustrate time-dependent changes and periodic patterns in graft-associated signals during follow-up.

**Figure 7:**
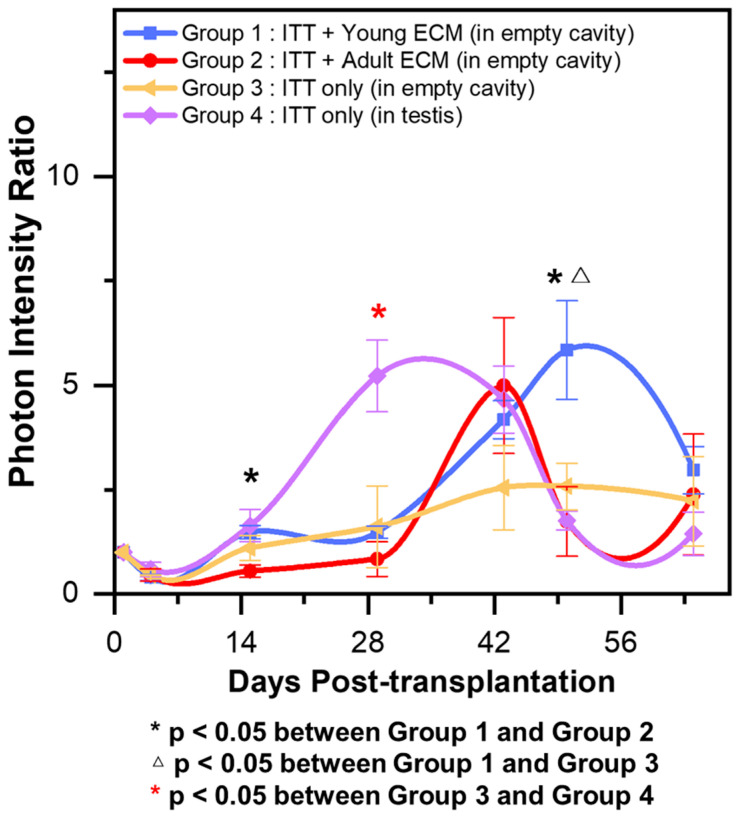
BLI photon intensity ratio over time. The normalized photon intensity ratio is plotted for all four groups over the post-transplantation follow-up period. Data are shown as mean ± SEM, with *n* = 3 animals per group. A black asterisk denotes a significant difference between Group 1 (ITT plus young dT-ECM) and Group 2 (ITT plus adult dT-ECM). A triangle denotes a significant difference between Group 1 and Group 3 (ITT only in the empty cavity). A red asterisk denotes a significant difference between Group 3 and Group 4 (ITT injected into the intact testis). Statistical significance was defined as *p* < 0.05.

Longitudinal bioluminescence imaging (BLI) was used to monitor graft survival and growth after transplantation. Across groups, BLI signals showed a periodic pattern over time, and the timing of peak signals differed between groups, suggesting dynamic changes in graft activity during follow-up (Figure 6 and Figure 7). These changes may be influenced by cell survival, proliferation, vascularization-supported viability, and metabolic activity.

Among the cavity implantation groups, Group 3 (ITT transplanted into the empty testicular cavity without dT-ECM) did not show a distinct growth peak, with BLI intensity only slightly higher than that on day 1. In contrast, Group 1 (ITT co-transplanted with young dT-ECM) exhibited a delayed but clear peak at approximately day 49. At day 49, the BLI signal in Group 1 was significantly higher than that in Group 3, indicating that adding dT-ECM was associated with more sustained support and greater graft stability over time. Group 2 (ITT co-transplanted with adult dT-ECM) showed a weaker response than Group 1, suggesting that the donor age of dT-ECM may influence graft outcomes.

Group 4, in which ITT was injected into the intact recipient testis, showed the earliest prominent BLI peak at day 28. Although the underlying mechanism cannot be determined from BLI alone, this earlier peak may be consistent with the rapid establishment of a supportive local environment within the intact testis. The subsequent decline in Group 4 may reflect, at least in part, physical constraints imposed by the limited available space when the recipient testicular tissue is preserved.

Overall, the periodic changes in BLI signals likely reflect a combination of intrinsic growth dynamics of the graft and extrinsic factors such as the available implantation space and local microenvironment.

### 2.4. Spermatogenesis and Mature Sperm Were Seen Among Groups 65 Days After Transplantation

**Figure 8:**
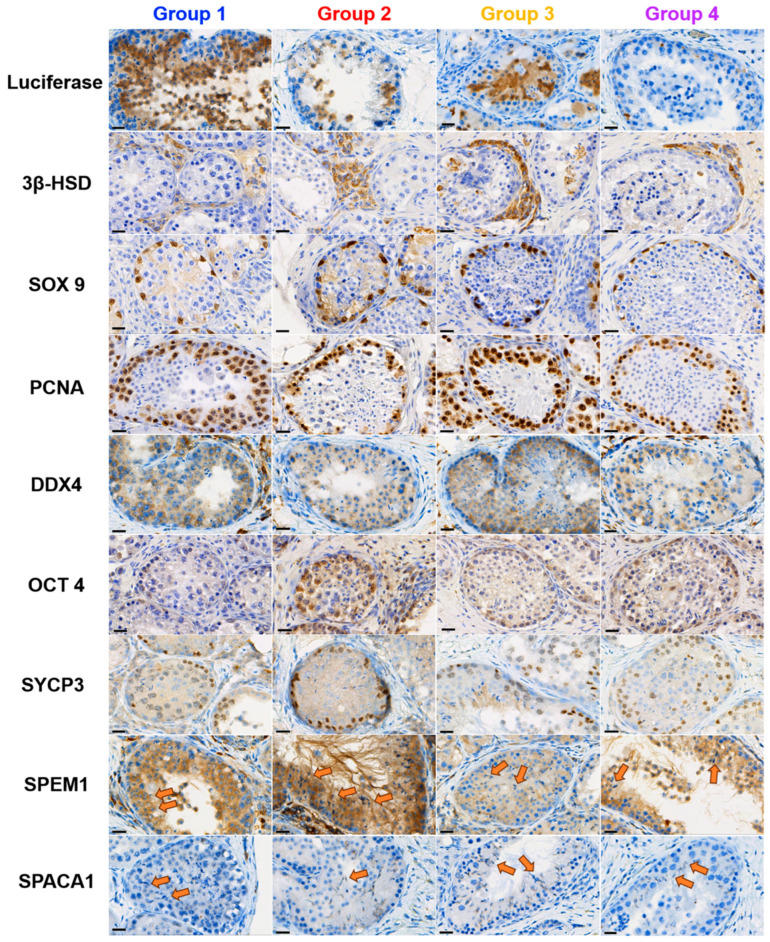
Immunohistochemical analysis of spermatogenesis at day 65 after transplantation. Representative IHC images of recovered grafts from Groups 1 to 4 harvested at day 65 are shown to assess spermatogenic progression. The panels represent separate sections or fields and illustrate the presence of stage-associated spermatogenic markers, rather than co-localization within the same tubules. Donor-derived cells are identified by luciferase staining. Somatic cell components are indicated by 3β-HSD for Leydig cells and SOX9 for Sertoli cells. Germ cell markers include OCT4 for spermatogonial stem or progenitor cells, PCNA for proliferating cells, and SYCP3 for meiotic spermatocytes. Markers of late-stage spermatogenesis include SPEM1 and SPACA1. These staining results provide qualitative evidence of spermatogenic progression across groups. Orange arrows indicate sperm-like cells. Bar = 20 μm.

At approximately 9 weeks after transplantation, on day 64 or 65, grafts from all groups were harvested for histological and immunohistochemical evaluation to assess spermatogenesis (Figure 8). Donor-derived somatic cells and germ cells remained detectable in all recovered grafts from Groups 1 through 4. Consistently, IHC confirmed the presence of key somatic cell populations, including 3β HSD-positive Leydig cells and SOX9-positive Sertoli cells, indicating the maintenance of essential cellular components of the testicular niche. Germ cell markers spanning multiple developmental stages were also observed, including OCT4 for spermatogonial stem or progenitor cells, PCNA for proliferating cells, and SYCP3 for meiotic spermatocytes. Importantly, mature spermatogenesis was evident in all groups, with the presence of mature sperm supported by positive staining for SPEM1, a marker of spermatid maturation, and SPACA1, an acrosomal and sperm-associated marker. These staining results provide qualitative evidence of advanced spermatogenic stages, including spermatids and sperm-like cells, across groups by day 65, although they were not designed for quantitative comparisons between groups.

## 3. Discussion

The results of this study highlight several factors that are associated with the long-term stability and functional progression of ITTs. In particular, our data emphasize the quality and biological function of dT-ECM, the donor age of the scaffold, and the local transplantation niche. Together, these observations provide a basis for refining ITTT strategies.

First, before interpreting the biological effects of dT-ECM, the quality of decellularization should be considered. A commonly cited benchmark for decellularization is residual dsDNA content below 50 ng/mg dry ECM, DNA fragment length below 200 bp, and absence of visible nuclear material. In the present study, residual DNA content remained above the 50 ng/mg threshold. However, because dT-ECM samples were extremely small and lightweight, dry-weight normalization may be sensitive to minor weighing variability. Therefore, decellularization efficiency was interpreted using multiple complementary assessments. The protocol removed 98.65–99.34% of DNA, with no obvious residual cellular material observed by histology or SEM, preservation of key ECM components, and no apparent adverse graft response after transplantation. These findings support highly efficient decellularization, although further optimization may be required to meet all conventional quantitative benchmarks.

Second, the presence of dT-ECM was associated with improved long-term graft stability. Co-transplantation of ITT with young dT-ECM into the recipient testicular cavity, compared with ITT alone, produced a significantly higher and more sustained BLI signal, with a clearer peak emerging later at approximately day 49. This pattern suggests that dT-ECM may function as a bioactive scaffold rather than merely serving as a space-filling material. By preserving tissue-specific matrix architecture and components such as collagens, laminins, and other ECM molecules, dT-ECM may provide a structural framework and may potentially support revascularization, nutrient delivery, and biochemical signaling that help maintain graft viability over time. These roles are consistent with the broader functions of the ECM [11,12,13]. In the testis, ECM proteins such as laminins and collagens regulate cellular interactions and guide germ cell differentiation and spermatogenesis [14], which may present a possible hypothesis for how dT-ECM contributes to improved graft performance in this study.

Third, scaffold donor age also appeared to potentially influence the transplantation outcomes. ITT co-transplanted with young dT-ECM from young donors showed a more sustainable response than ITT co-transplanted with adult dT-ECM. This finding may indicate that the ECM undergoes meaningful changes during maturation that can affect its supportive capacity. Many reports have shown that donor age can influence graft performance and spermatogenesis, with younger donors generally providing better outcomes than older donors [15,16,17,18]. In contrast, very few studies have examined how donor age affects the use of ECM-based scaffolds. To our knowledge, this is the first study to propose the hypothesis that the donor age of the dT-ECM may influence ITTT outcomes. The observed age-dependent difference should therefore be interpreted as a hypothesis-generating observation requiring further validation. Although the difference in seminiferous tubule diameter indicates age-related architectural variation, its functional relevance remains unclear. Whether these structural differences correspond to changes in stiffness, porosity, or diffusion properties was not directly evaluated in the present study and should be addressed in future biophysical studies. Also, developmental differences in ECM hydration, collagen organization, cross-linking, and overall compliance have been reported across tissues and may alter the mechanical environment experienced by immature testicular cells and spermatogonial stem cells (SSCs). This concept is consistent with prior work indicating that SSC proliferation and differentiation change as the testis develops from perinatal to pubertal stages [19,20]. Another relevant consideration is that regulation of the stem cell niche is age-dependent [21], raising the possibility that mature ECM may provide signals that are less aligned with the requirements of immature graft tissue.

Potential molecular mechanisms may also contribute to the observed age-dependent differences between young and adult dT-ECM, although this requires future validation. Because the present study did not include direct molecular profiling, these mechanisms remain speculative. Age-related variation in matrix proteins, glycosaminoglycans, and heparan sulfate proteoglycans (HSPGs) can alter how growth factors are retained, concentrated, and presented to germ cells and supporting somatic cells [22]. HSPGs embedded in the ECM can modulate the bioavailability of key growth factors [23], including germline niche-related factors such as GDNF and FGF2 [24,25,26]. Further molecular and biophysical characterization will be needed to determine whether these potential mechanisms contribute to the observed difference in graft performance.

Fourth, the transplantation space and local niche were also associated with distinct graft growth dynamics. Many studies have shown that orthotopic transplantation yields better outcomes than ectopic transplantation [27], but few have examined how the available space at the orthotopic site relative to the graft size influences transplantation outcome. The possible mechanisms contributing to these results are the mechanical threshold, vascular infiltration, and paracrine priming [28,29]. The direct injection of ITT into the intact recipient testis resulted in the earliest bioluminescence peak, which is compatible with immediate access to a pre-existing vascular network and resident supporting cells. This intraparenchymal environment may offer paracrine signals from neighboring host cells that likely accelerate the initial maturation of the transplanted fragments. However, the signal in this group subsequently declined in contrast to the more sustained activity observed in the groups that used a cleared testicular cavity. One interpretation is that the graft eventually reaches a mechanical threshold within the host testis. Previous studies in other tissues have suggested that confined tissue expansion and increased mechanical stress can impair local blood flow and tissue growth [30,31]. In the testis, the tunica albuginea is a dense connective tissue capsule that may limit tissue expansion as graft volume increases. Although intratesticular pressure and the mechanical properties of the tunica albuginea were not directly measured in the present study, it is possible that spatial limitation within the intact testis contributed to the later decline in graft activity. Moreover, studies have shown that the physical pressure can also influence the interstitial fluid pressure, which in turn modulates biological responses such as transcapillary exchange and the targeted transport of hormones and nutrients [32,33]. In contrast, the lack of an early growth peak in the empty cavity groups may suggest that a vacant space lacks the necessary biological priming required for rapid development. The initial delay in vascular infiltration and the absence of host-derived signaling molecules likely prevent the graft from achieving the early growth velocity. These observations suggest that a successful transplantation strategy depends on a balance between immediate biological support and sufficient physical space for maturation.

A key advantage of this study is the establishment of an in vivo platform combining decellularized testicular matrix with longitudinal BLI. Unlike traditional histological snapshots, BLI allows for the continuous, non-invasive monitoring of graft activity within the same living host. This approach uniquely identifies dynamic growth peaks and kinetic shifts across different transplantation niches. Furthermore, this work is the first to demonstrate that scaffold donor age is a critical variable, proving that young dT-ECM provides a biologically superior niche compared to adult matrix. By integrating real-time in vivo monitoring with site-specific scaffolds, this study offers a deeper understanding of the biomechanical requirements for successful tissue maturation.

Several limitations should be considered when interpreting these findings. First, BLI provides an indirect measure of graft viability and proliferation and does not by itself establish complete functional fertility. While histology and IHC at day 65 supported spermatogenic progression and the presence of mature sperm-like cells, no functional fertility testing was performed in this study. Therefore, additional endpoints are required to evaluate sperm quality, functional competence, fertilization capacity, offspring generation, and genetic integrity. Second, ECM preservation was assessed qualitatively rather than quantitatively. The retention level of key ECM components was not quantified. Because decellularization caused marked tissue shrinkage and collapse of the original tubular architecture, image-based quantification may not reliably represent true ECM retention. Future studies are needed to determine the extent of ECM retention and clarify how preserved matrix components contribute to graft outcomes. Third, although young dT-ECM performed better than adult dT-ECM in this study, the specific molecular and mechanical features responsible for this difference were not defined. Proteomic profiling, quantitative assessment of growth factor-binding capacity, and mechanical testing would help clarify which properties are most strongly linked to graft stability and maturation. Fourth, decellularization conditions can affect matrix ultrastructure and bioactive factor retention. Finally, this study was conducted in a mouse model, and the findings may not fully reflect the biological, anatomical, and translational challenges of human immature testicular tissue transplantation. Further optimization and standardization will be important to ensure reproducibility across batches and to define how processing choices shape scaffold performance.

## 4. Materials and Methods

### 4.1. Animals

The donor animals were FVB/N-Tg(*PolII-luc*) Ltc transgenic mice (hemizygotic for a construct comprising the mouse RNA polymerase II promoter and modified firefly luciferase cDNA), generated by the microinjection of the *PolII-luc* transgene into fertilized FVB/N embryos, and maintained as in-house breeding colonies by technical service from Level Biotechnology Inc. (New Taipei City, Taiwan). Offspring were identified by ear punches and verification of tissue bioluminescence intensity under BLI to confirm hemizygotic FVB/N-Tg(*PolII-luc*) Ltc transgenic status [34]. Recipient mice were age-matched FVB/NJNarl wild-type animals purchased from either the National Laboratory Animal Center (NLAC, Taipei, Taiwan) or LASCO Biotechnology. All animals were raised under specific pathogen-free (SPF) conditions in the animal facilities of Taipei Medical University, maintained at 22–24 °C, with a 12-h light/dark cycle and unrestricted access to food and water. All procedures were reviewed and approved by the Animal Experimental Committee of Taipei Medical University and complied with the Guide for the Care and Use of Laboratory Animals.

### 4.2. Manufacture of Decellularized Testicular Extracellular Matrix Scaffold (dT-ECM)

We prepared decellularized testicular extracellular matrix (dT-ECM) from syngeneic male FVB mouse testes according to a published protocol [5]. To assess whether donor age affects transplantation outcomes, dT-ECM was generated from the testes of 3-week-old (young) and 10~14-week-old (adult) mice to compare young versus adult ECM. Both dT-ECM types were prepared using the same procedure.

Briefly, mice were euthanized and intact testes with the tunica albuginea were harvested. After a brief rinse in distilled deionized water (ddw) to remove residual body fluids and blood, the testes underwent 3 freeze–thaw cycles at −20 °C/−80 °C/37 °C, with incubation for >8 h at −20 °C and −80 °C and 1 h at 37 °C. The testes were then rinsed again in ddw and perforated along 3 axes using needles (adult: 18 G; young: 30 G; TERUMO^®^, Tokyo, Japan) to enhance subsequent detergent penetration. Samples were immersed in ddw and sonicated (KJ-1360AL, Shenzhen, China) for 5 min, repeated 3 times with ddw replacement.

Decellularization was performed by incubation in 1% SDS (SDS101000, BIOMAN, New Taipei City, Taiwan) on a tube rotator (R4045 BENCHMARK, Las Vegas, NV, USA) at 60 rpm for 2 days, followed by 1% Triton™ X-100 (WXBD9064V, Sigma, Santa Louis, MO, USA) for an additional 2 days. Solutions were replaced 2 times daily, and samples were sonicated for 5 min before each solution change. Finally, tissues were extensively washed with ddw for at least 7 days to remove residual SDS and Triton X-100.

### 4.3. Characterization of the Decellularized Testicular Extracellular Matrix Scaffold (dT-ECM)

To verify effective decellularization and preservation of key extracellular matrix components, residual DNA in dT-ECM was quantified using the EasyPure Genomic DNA Spin Kit (GT100; BIOMAN, New Taipei City, Taiwan). In addition, dT-ECM samples were paraffin-embedded, sectioned at 5 µm, deparaffinized, and stained with hematoxylin and eosin (H&E), Masson’s trichrome, and Alcian blue to evaluate overall matrix architecture and the presence of collagen and glycosaminoglycans (GAGs), as previously described [35]. For immunohistochemistry (IHC), 5 µm Bouin-fixed paraffin sections were deparaffinized in xylene and rehydrated through graded ethanol (100%, 100%, 80%, 60%, 50%). Heat-induced antigen retrieval was performed using Target Retrieval Solution (K805; Dako, Santa Clara, CA, USA) by incubating slides at 63 °C for 60 min, followed by cooling at room temperature for ≥1 h. After blocking with Dako Blocking Solution (S2023, Santa Clara, CA, USA) for 5 min, sections were incubated with anti-collagen I (AF7001), anti-collagen IV (AF0510), anti-fibronectin (AF5335), and anti-laminin 2α (DF13275) antibodies (Affinity Biosciences, Changzhou, China) diluted in Antibody Diluent (S3022; Dako, Santa Clara, CA, USA) vernight at 4 °C. Signals were detected using an HRP-conjugated secondary antibody and developed with DAB (K5007; Dako, Santa Clara, CA, USA), followed by H&E counterstaining. Whole-slide images were acquired using a slide scanner MoticEasyScan Pro 6 with Motic DSAssistant software (Motic China; Xiamen, China) and staining was evaluated under identical scanning and analysis settings across groups.

### 4.4. Transplantation Procedure and Grouping

This study followed the experimental design and transplantation procedures described in our previous report [36]. Three-week-old FVB/N-Tg(*PolII-luc*)Ltc transgenic male mice were used as donors of immature testicular tissue, and age-matched wild-type FVB/N male mice served as isograft recipients. One testis from each donor was harvested, and the tunica albuginea was removed. The average weight of each testis was 41.70 ± 6.71 mg (mean ± SD). Each whole donor testis was minced into small ITT fragments with an average size of approximately 0.29 mm × 0.12 mm [37]. The ITT fragments were washed with DPBS in a centrifuge tube and allowed to settle for several minutes. After removing the supernatant, approximately 20 μL of ITT fragments was obtained from each donor testis. This 20 μL ITT fragment suspension, corresponding to one 3-week-old donor testis, was transplanted according to the assigned experimental group. For designated groups, one recipient testis was surgically removed, and donor ITT, together with the prepared dT-ECM, was implanted into the recipient’s testicular cavity according to group assignment. The incision was then closed with sutures.

The study included four groups. Group 1: ITT co-transplanted with young dT-ECM into the recipient testicular cavity. Group 2: ITT co-transplanted with adult dT-ECM into the recipient testicular cavity. Group 3: ITT transplanted alone into the recipient testicular cavity (no dT-ECM). Group 4: The recipient testis was not removed. Instead, ITT was directly injected into the intact testis without dT-ECM. In all groups, the amount of ITT transplanted corresponded to the tissue obtained from one 3-week-old donor testis.

### 4.5. Bioluminescence Imaging (BLI) for Longitudinal Tracking of Testicular Tissue Grafts

The engrafted testicular tissues were longitudinally monitored using bioluminescence imaging (BLI) with the IVIS XRMS (PerkinElmer, Hopkinton, MA, USA). Luminescent signals were quantified with Living Image software (version 4.7.3, PerkinElmer, Hopkinton, MA, USA). Nude or shaved-skin mice were used to minimize autofluorescence interference caused by fur. To ensure imaging consistency, the abdominal skin of recipient mice was shaved 2 h prior to each session. D-luciferin potassium salt (150 mg/kg; L-8220, Cas No. 115144-35-9, Biosynth, Compton, UK) was administered intraperitoneally 10 min before imaging. Mice were placed in a light-tight chamber under continuous 2% isoflurane anesthesia, and images were acquired from the ventral side at a field of view of 20 cm for 3 min under high-resolution mode. A cooled charge-coupled device (CCD) camera captured the bioluminescent signal, and overlay images were simultaneously recorded using a white-light illumination mode within the same chamber.

Photon emission was quantified by summing the pixel intensities within a defined region of interest (ROI; 1.5 × 1.5 cm, covering the whole transplanted region). Absolute light intensity was calibrated using an 8-inch integrating sphere (OL Series 425 Variable Low-Light-Level Calibration Standard; Optronic Laboratories, Orlando, FL, USA) as previously described [38]. The photon counts generated by the luciferase-catalyzed oxidation of luciferin were converted to physical radiance units (photons/s/cm^2^/sr) [39].

BLI data were recorded one day after transplantation as baseline, followed by approximately weekly imaging sessions. Minor deviations of ±1–2 days were allowed, depending on instrument availability. To account for interindividual variation among recipient mice, the luminescent intensities were normalized by dividing each subsequent photon value by the signal obtained on the first day post-transplantation. This normalization generated a photon intensity ratio that reflected the relative increase or decrease in graft activity over time.

### 4.6. Scanning Electron Microscopy (SEM)

The morphology and ultrastructure of the dT-ECM were examined using a scanning electron microscope (SEM; SU3500, Hitachi, Tokyo, Japan). The dehydrated specimens were then dried using a critical-point dryer, mounted on aluminum stubs, and sputter-coated with a thin layer of gold to enhance conductivity. The surface topography was observed at an accelerating voltage of 15 kV and a magnification of 500× to evaluate the fibrillar architecture and porosity of the decellularized testicular ECM.

### 4.7. Immunohistochemistry Analyses

At 65 days after transplantation, recipient mice were euthanized, and grafted testicular tissues from the study groups were harvested for histological and immunohistochemical analyses. Grafts had been transplanted with or without a scaffold. Collected tissues were fixed in Bouin’s solution (HT10132, Sigma Aldrich, Saint Louis, MO, USA) overnight, embedded in paraffin wax, sectioned at 5 µm, deparaffinized, and stained with H&E. Random sections from each recipient were examined for evidence of spermatogenesis, including the presence of germ cells at different developmental stages, meiotic figures, and spermatozoa.

Immunohistochemistry was performed according to a previously described protocol [40]. Briefly, 5 µm Bouin-fixed paraffin sections were deparaffinized in xylene and rehydrated through graded ethanol (100%, 100%, 80%, 60%, 50%), rinsed in distilled water, and incubated in Target Retrieval Solution (K805, Dako, Santa Clara, CA, USA). Slides were heated in an oven at 63 °C for 60 min and then cooled at room temperature for at least 1 h. After rinsing with phosphate-buffered saline, nonspecific binding was blocked using Blocking Solution (S2023, Dako, Santa Clara, CA, USA) for 5 min. Sections were incubated overnight at 4 °C in Antibody Diluent (S3022, Dako, Santa Clara, CA, USA) with rabbit polyclonal antibodies against firefly luciferase (ab185924, AbCam, Cambridge, UK), SOX9 (ab185966, AbCam Inc., Cambridge, UK), OCT4 (ab181557, AbCam Inc., Cambridge, UK), PCNA (10205-2-AP, Proteintech, Rosemont, IL, USA), SPACA1 (12829-1-AP, Proteintech Group, Inc, Rosemont, IL, USA), DDX4 (51042-1-AP, Proteintech Group, Inc, Rosemont, IL, USA), 3β-HSD (A1823, Abclonal, Woburn, MA, USA), SYCP3 (NB300-232, Novus Biologicals, Centennial, CO, USA), and SPEM1 (PA5-144156, Thermo Fisher Scientific, Waltham, MA, USA). Sections were then incubated with an HRP-conjugated secondary antibody, developed using a DAB detection system (K5007, Dako, Santa Clara, CA, USA), and counterstained with H&E. Staining was evaluated using a slide scanner MoticEasyScan Pro 6 with Motic DSAssistant software (Motic China; Xiamen, China).

### 4.8. Statistical Analysis

The SAS package (version 9.4; SAS Institute, Cary, NC, USA) was used for all data analyses. The Mann–Whitney U test was used to compare the photon yields between the four groups. A two-tailed *t* test was used for the statistical analysis of BLI intensity, and *p* < 0.05 was considered statistically significant.

## 5. Conclusions

In conclusion, the present study supports the utility of dT-ECM as a scaffold that promotes the long-term stability of ITT grafts and indicates that both scaffold donor age and the transplantation niche are key variables that influence graft dynamics. Decellularized testicular ECM functions as a bioactive scaffold that retains essential structural and biochemical cues. Donor age emerges as a critical parameter. The young matrix group appeared to provide more sustained support for spermatogenic progression, as reflected by a more stable increase and a prolonged spermatogenic peak over time. A comparison of different transplantation settings further shows that an intact host testis can drive rapid early graft growth but ultimately imposes mechanical constraints that limit sustained maturation, whereas a cleared cavity offers greater space for later tissue expansion. These observations highlight that successful fertility restoration requires a balance between immediate biological support and sufficient physical space. Future work that combines detailed scaffold characterization with long-term functional assessment will be important to define optimized scaffold formulations, including young ECM-derived carriers, and to determine how best to provide appropriate biochemical and mechanical support within an implantation environment that permits full tissue maturation. Further studies are required to establish functional fertility, long-term safety, and clinical applicability.

## Data Availability

All data generated in the study are presented in the manuscript.

## Abbreviations

TTC: testicular tissue cryopreservation; SSCT: spermatogonial stem cell transplantation; IVS: in vitro spermatogenesis; ITTT: immature testicular tissue transplantation; ECM: extracellular matrix; PLLA: poly(L-lactic acid); dT-ECM: decellularized testicular extracellular matrix; NLAC: National Laboratory Animal Center; SPF: specific pathogen-free; ddw: distilled deionized water; SDS: sodium dodecyl sulfate; H&E: hematoxylin and eosin; GAGs: glycosaminoglycans; IHC: immunohistochemistry; ITT: immature testicular tissue; BLI: bioluminescence imaging; CCD: cooled charge-coupled device; ROI: region of interest; SEM: scanning electron microscopy; 3β-HSD: 3β-hydroxysteroid dehydrogenase; SOX9: SRY-box transcription factor 9; OCT4: octamer-binding transcription factor 4; PCNA: proliferating cell nuclear antigen; SPACA1: sperm acrosome-associated 1; DDX4: DEAD-box helicase 4; SYCP3: synaptonemal complex protein 3; SPEM1: spermiogenesis associated factor (or marker of spermatid maturation); SSCs: spermatogonial stem cells; HSPGs: heparan sulfate proteoglycans; GDNF: glial cell line-derived neurotrophic factor; bFGF: basic fibroblast growth factor.
